# Supplementary material for: Oral health status, oral hygiene behaviors, and caries risk assessment of individuals with special needs: a comparative study of Pakistan and Saudi Arabia
Source: PeerJ. 2025 May 8;13:e19286. doi: 10.7717/peerj.19286 (PMC12066100; doi:10.7717/peerj.19286)
Supplement: Supplemental Information 2 [file peerj-13-19286-s002.doc]

**Completed STROBE checklist**

This checklist was elaborated using formal items recommended for cross-sectional studies from STROBE statement (https://www.strobe-statement.org).

|  | Item No | Recommendation | Respected ? | Comments and quotes | |
| --- | --- | --- | --- | --- | --- |
| **Title and abstract** | 1 | (*a*) Indicate the study’s design with a commonly used term in the title or the abstract | Yes | | Study design is indicated in the Methods section of the abstract (Page 3, line 51)  “This was cross-sectional study design” |
|  |  | (*b*) Provide in the abstract an informative and balanced summary of what was done and what was found | Yes | | This information are stated in the study abstract (study objective described, method and results described) (Page 3, line 51-60) |
| Introduction | | |  | |  |
| Background/rationale | 2 | Explain the scientific background and rationale for the investigation being reported | Yes | | Rationale and existing literature are stated in the introduction section, (Page 4,5 , line 76-124) |
| Objectives | 3 | State specific objectives, including any prespecified hypotheses | Yes | | A statement at the end of the introduction specifies the specific goals and objectives. (Page 6, line 125-132) |
| Methods | | |  | |  |
| Study design | 4 | Present key elements of study design early in the paper | Yes | | Study design is stated in the first subsection of Methods. Key elements are all described in the methods. (Page 7, line 135-155)  This was cross-sectional study design |
| Setting | 5 | Describe the setting, locations, and relevant dates, including periods of recruitment, exposure, follow-up, and data collection | Mostly | | Setting, contexts, dates of inclusion, are fully described in the method section . (Page 7, line 135-155)  ‘The study focused on disabled individuals, with participants recruited from the Institute of Special Education and Bahria College for Special Needs in Islamabad, Pakistan, and the Saudi Institute of Rehabilitation Medicine in Jouf, Saudi Arabia’ |
| Participants | 6 | (*a*) Give the eligibility criteria, and the sources and methods of selection of participants | Yes | | Study population is described is the method section, as well as selection criteria. (Page 7, line 139-145)  “. The study population included teenagers, adults and young aged 13 years and older with hearing, visual, and intellectual disabilities. Participants with other types of disabilities (autism, cerebral palsy, or other neurological and developmental disorders) and those whose parents or guardians declined to consent were excluded” |
| Variables | 7 | Clearly define all outcomes, exposures, predictors, potential confounders, and effect modifiers. Give diagnostic criteria, if applicable | Yes | | Standardized variable definitions were used across all programs, which are presented in method section. (Page 7) |
| Data sources/ measurement | 8* | For each variable of interest, give sources of data and details of methods of assessment (measurement). Describe comparability of assessment methods if there is more than one group | Yes | | Data collection and measurement was the same for all variables, and is described in the methods section. (Page 8, line 161-197)  “Data were collected through a valid questionnaire’ |
| Bias | 9 | Describe any efforts to address potential sources of bias | Yes | | We notably tried to reduce bias by excluding incomplete questionnaires. The analysis section also explains this part. (page 10, line 205) |
| Study size | 10 | Explain how the study size was arrived at | Yes | | The method describes the sample size estimation for this study (Page 7, line 143-155). |
| Quantitative variables | 11 | Explain how quantitative variables were handled in the analyses. If applicable, describe which groupings were chosen and why | Yes | | Definitions of all categories for variables are presented method section. (Page 9, line 183-200)  “The first part of the questionnaire collected the sociodemographic information (age and gender etc). second part consist of Interprofessional Learning Scale (RIPLS) questionnaire. This 19-item self-report instrument comprises four subscales: teamwork and collaboration (TC), negative professional identity (NPI), positive professional identity (PPI), and roles and responsibilities (RR). |
| Statistical methods | 12 | (*a*) Describe all statistical methods, including those used to control for confounding | Yes | | (Page 10, line 205-209)  “Descriptive statistics were computed to summarize the data, with frequency distributions for categorical variables. Chi-square tests were used to assess the association between oral health indicators and sociodemographic factors. Binary logistic regression analysis was employed to explore relationships between caries risk categories and sociodemographic variables. All statistical analyses were performed using IBM SPSS (version 25.0)**,** with a significance level set at p < 0.05.’ |
|  |  | (*b*) Describe any methods used to examine subgroups and interactions | Yes | | (Page 10, line 205) |
|  |  | (*c*) Explain how missing data were addressed | Yes | | This is described in the method section. (Page 10, line 207)  “Missing values were not inferred” |
|  |  | (*d*) If applicable, describe analytical methods taking account of sampling strategy | N/A | | Non applicable |
|  |  | (*e*) Describe any sensitivity analyses | N/A | | Non applicable |
| Results | | |  | |  |
| Participants | 13* | (a) Report numbers of individuals at each stage of study—eg numbers potentially eligible, examined for eligibility, confirmed eligible, included in the study, completing follow-up, and analysed | Yes | | This is described at the beginning of result section. (page 10.line 112)  “A total of 189 participants with disabilities were included in the study” |
|  |  | (b) Give reasons for non-participation at each stage | Yes | | This is described at the beginning of result section. (page 10 .line 112)  A total of 189 participants with disabilities were included in the study. |
|  |  | (c) Consider use of a flow diagram | N/A | | Use of a flow diagram was not deemed appropriate. |
| Descriptive data | 14* | (a) Give characteristics of study participants (eg demographic, clinical, social) and information on exposures and potential confounders | Yes | | Table 1 describes the participants and programs included.  “Table 1: Socio-demographic characteristics of the participants. |
|  |  | (b) Indicate number of participants with missing data for each variable of interest | Yes | | The total numbers of recorded data for each variable are stated in variable headline of each table |
| Outcome data | 15* | Report numbers of outcome events or summary measures | Yes | | All numbers are reported in Tables. |
| Main results | 16 | (*a*) Give unadjusted estimates and, if applicable, confounder-adjusted estimates and their precision (eg, 95% confidence interval). Make clear which confounders were adjusted for and why they were included | N/A | | N/A |
|  |  | (*b*) Report category boundaries when continuous variables were categorized | Yes | | Category boundaries are displayed in variable headings in the tables, where applicable (age groups) |
|  |  | (*c*) If relevant, consider translating estimates of relative risk into absolute risk for a meaningful time period | N/A | | N/A |
| Other analyses | 17 | Report other analyses done—eg analyses of subgroups and interactions, and sensitivity analyses | N/A | | N/A |
| Discussion | | |  | |  |
| Key results | 18 | Summarise key results with reference to study objectives | Yes | | Key results are described at the beginning of discussion section (page 12, line 261), and later on by the mean of a paragraph displaying main operational implications. They also are summarized in the conclusion |
| Limitations | 19 | Discuss limitations of the study, taking into account sources of potential bias or imprecision. Discuss both direction and magnitude of any potential bias | Yes | | Description of limitations is done at the end of discussion section.  (page 15.line 325-336) |
| Interpretation | 20 | Give a cautious overall interpretation of results considering objectives, limitations, multiplicity of analyses, results from similar studies, and other relevant evidence | Yes | | References were added where possible and discussed. Limitations were taken into account in the discussion. (page 15,16) |
| Generalisability | 21 | Discuss the generalisability (external validity) of the study results | Yes | | Study generalizable described in the discussion section. (Page 15, line 270-281) |
| Other information | | |  | |  |
| Funding | 22 | Give the source of funding and the role of the funders for the present study and, if applicable, for the original study on which the present article is based | Yes | | Funding information were displayed upon submission but not included in the manuscript, as requested. |
|  | | |  | |  |

*Give information separately for exposed and unexposed groups.
