# Supplementary material for: Oral health status, oral hygiene behaviors, and caries risk assessment of individuals with special needs: a comparative study of Pakistan and Saudi Arabia
Source: PeerJ. 2025 May 8;13:e19286. doi: 10.7717/peerj.19286 (PMC12066100; doi:10.7717/peerj.19286)
Supplement: Supplemental Information 3 [file peerj-13-19286-s003.docx]

**Survey Questionnaire of Oral health status, oral hygiene behaviors, and Caries Risk Assessment of Individuals with Special Needs**

Please complete the following questionnaire first two section by placing a Cross in the appropriate box.

**Section One: Socio-demographic Data:**

1. **Gender**

Male Female

1. **Age:** Exact age_______

13-16 17-26

27 and above

1. **Parent’s level of education**

No formal education School attended

College attended University attended

1. **Parent’s Occupation**

Labour Govt. Employee

Professional Others

1. **Length of employment (years)**

<5 5-10

- 1. 16 and above

1. **Disability of individuals**

Hearing loss Visual Impairment

Intellectual Disability/Down syndrome

**Section Two: Oral Hygiene behaviors/Knowledge:**

1. **How many times do you brush your teeth**
2. Once a day b. Twice

c. Thrice d. Do not brush

1. **Do you think oral health has an effect on general health?**
2. Yes B. No
3. **Do you think proper tooth brushing helps in maintaining oral hygiene**
4. Yes B. No
5. **Do you think carbonated drinks have adverse effect on teeth?**
6. Yes B. No
7. **Do you think sugary/sticky food items can damage teeth?**
8. Yes B. No
9. **Do you know how should teeth be cleaned?**
10. Finger b. stick c. Tooth-brush d. charcoal
11. **How long should you clean your teeth for?**
12. Less than a minute b. 2 min c. 5 min d. I don’t know
13. **Which type of toothpaste should be used?**
14. Fluoride containing b. without fluoride c. I don’t know
15. **What do you know is the ideal time for brushing your teeth?**
16. In the morning b. at night c. after every meal

d. all of the above

1. **Did your primary care giver or school-teacher educate you regarding oral health and maintenance?**
2. Yes b. No
3. **Have you been to a dentist during the past years?**
4. Yes b. No
5. **From where did you get information on how to keep your mouth clean?**
6. Parents b. Teacher
7. **Are you able to brush independently?**
8. Yes b. No

| **Caries Risk Assessment Form (CAMBRA)** | | | |
| --- | --- | --- | --- |
| Patient information | | | |
| **Disease Indicators** | YES = CIRCLE | YES = CIRCLE | YES =CIRCLE |
| Visible cavities or radiographic penetration of the dentin |  |  |  |
| Radiographic approximal enamel lesions (not in dentin) |  |  |  |
| White spots on smooth surfaces |  |  |  |
| Restorations last 3 years |  |  |  |
|  |  |  |  |
| **Risk Factors (Biological predisposing factors)** |  |  |  |
| Visible heavy plaque on teeth |  |  |  |
| Frequent snack (> 3x daily between meals) |  |  |  |
| Deep pits and fissures |  |  |  |
| Recreational drug use |  |  |  |
| Inadequate saliva flow by observation |  |  |  |
| Saliva reducing factors (medications/radiation/systemic) |  |  |  |
| Exposed roots |  |  |  |
| Orthodontic appliances |  |  |  |
|  |  |  |  |
| **Protective Factors** |  |  |  |
| Lives/work/school fluoridated community |  |  |  |
| Fluoride toothpaste at least once daily |  |  |  |
| Fluoride toothpaste at least 2x daily |  |  |  |
| Fluoride mouthrinse (0.05% NaF) daily |  |  |  |
| Fluoride varnish in last 6 months |  |  |  |
| Chlorhexidine prescribed/used one week each of last 6 months |  |  |  |
| Xylitol gum/lozenges 4x daily last 6 months |  |  |  |
| Calcium and phosphate paste during last 6 months |  |  |  |
|  | **HIGH** | **MODERATE** | **LOW** |
| CARIES RISK ASSESSMENT (CIRCLE): **HIGH, MODERATE, LOW**  **(EXTREME RISK= HIGH RISK + SEVERE SALIVARY GLAND HYPOFUNCTION)** | | | |
