# Supplementary material for: Oral health status, oral hygiene behaviors, and caries risk assessment of individuals with special needs: a comparative study of Pakistan and Saudi Arabia
Source: PeerJ. 2025 May 8;13:e19286. doi: 10.7717/peerj.19286 (PMC12066100; doi:10.7717/peerj.19286)
Supplement: Supplemental Information 5 [file peerj-13-19286-s005.docx]

**Survey Questionnaire of Oral health status, oral hygiene behaviors, and Caries Risk Assessment of Individuals with Special Needs**

Please complete the following questionnaire first two section by placing a Cross in the appropriate box.

**SPSS data code is mentioned along with answer options in brackets ( )**

**Section One: Socio-demographic Data:**

1. **Gender**

Male (1) Female (2)

1. **Age:** Exact age_______

13-16 (1) 17-26 (2)

27 and above (3)

1. **Parent’s level of education**

No formal education (1) School attended (2)

College attended (3) University attended (4)

1. **Parent’s Occupation**

Labour (1) Govt. Employee (2)

Professional (3) Others (4)

1. **Length of employment (years)**

<5 (1) 5-10 (2)

- 1. (3) 16 and above (4)

1. **Disability of individuals**

Hearing loss (1) Visual Impairment (2)

Intellectual Disability/Down syndrome (3)

**Section Two: Oral Hygiene behaviors/Knowledge:**

1. **How many times do you brush your teeth**
2. Once a day (1) b. Twice (2)

c. Thrice (3) d. Do not brush (4)

1. **Do you think oral health has an effect on general health?**
2. Yes (1) B. No (2)
3. **Do you think proper tooth brushing helps in maintaining oral hygiene**
4. Yes (1) B. No (2)
5. **Do you think carbonated drinks have adverse effect on teeth?**
6. Yes (1) B. No (2)
7. **Do you think sugary/sticky food items can damage teeth?**
8. Yes (1) B. No (2)
9. **Do you know how should teeth be cleaned?**
10. Finger (1) b. stick (2) c. Tooth-brush (3) d. charcoal (4)
11. **How long should you clean your teeth for?**
12. Less than a minute (1) b. 2 min (2) c. 5 min (3) d. I don’t know (4)
13. **Which type of toothpaste should be used?**
14. Fluoride containing (1) b. without fluoride (2) c. I don’t know (3)
15. **What do you know is the ideal time for brushing your teeth?**
16. In the morning (1) b. at night (2) c. after every meal (3)

d. all of the above (4)

1. **Did your primary care giver or school-teacher educate you regarding oral health and maintenance?**
2. Yes (1) b. No (2)
3. **Have you been to a dentist during the past years?**
4. Yes (1) b. No (2)
5. **From where did you get information on how to keep your mouth clean?**
6. Parents (1) b. Teacher (2)
7. **Are you able to brush independently?**
8. Yes (1) b. No (2)

**Oral Examination by trained Dentist:**

Oral examination will be done using a mouth mirror and WHO probe to record Molar classification, DMFT score, Gingival index, Plaque index , visible plaque , simplified oral hygiene index and Community periodontal index.

**DMFT Score**

The caries experience is expressed as the total number of teeth that are decayed (D), missing (M) and Filled (F) teeth:

**
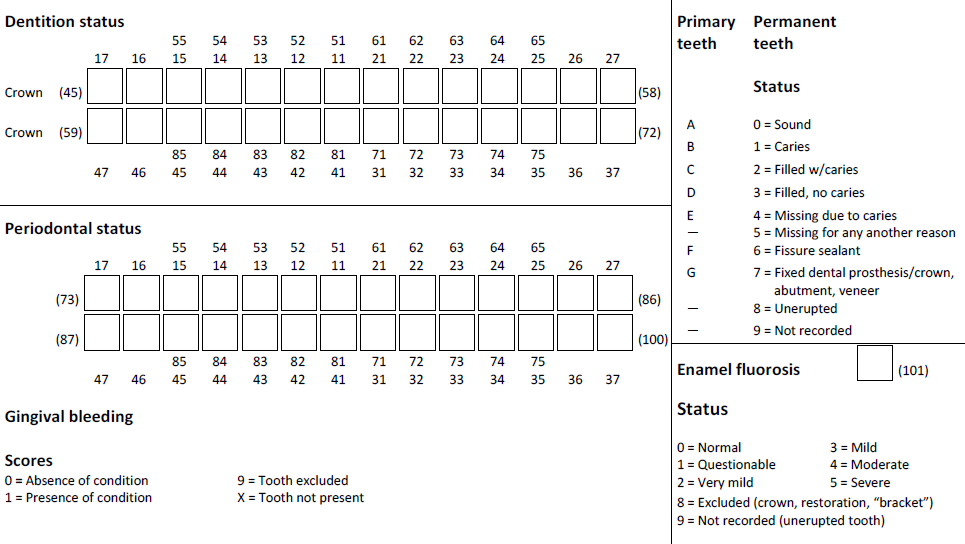
**

Total DMFT score (D+M+F) = ___________

**Gingival Index**

Gingival health will be assessed by using Loe & Sillness gingival index. Evaluated by examination of six index teeth ( molars and incisors) using Loe and Sillness Gingival Index. Each of the buccal, mesial, lingual and distal surfaces of the gingival tissues is given a score of 0-3. Scores from the 4 areas of the tooth are added and divided by four to give the GI for the tooth. Scores for individual teeth may be grouped to designate the GI for the group of teeth. The scores may be added and divided by the number of teeth examined to derive the GI for the individual.


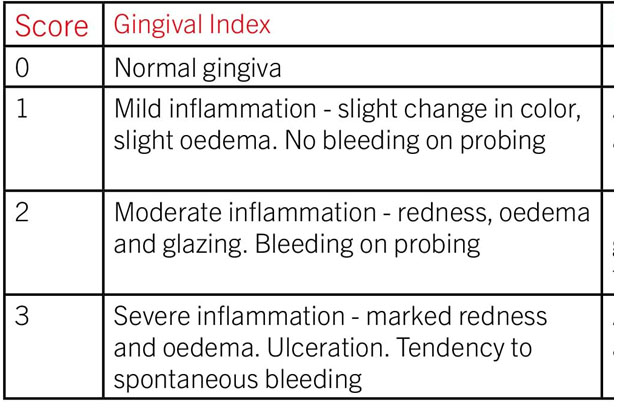

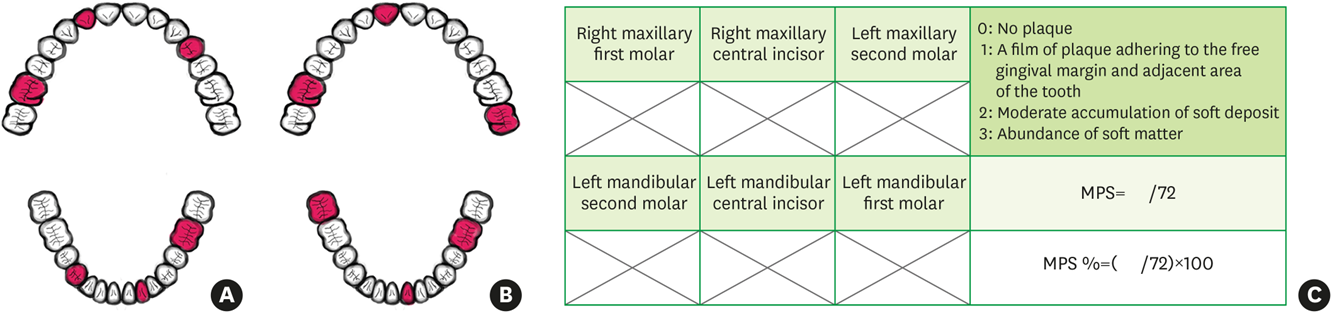


**SPSS data code**


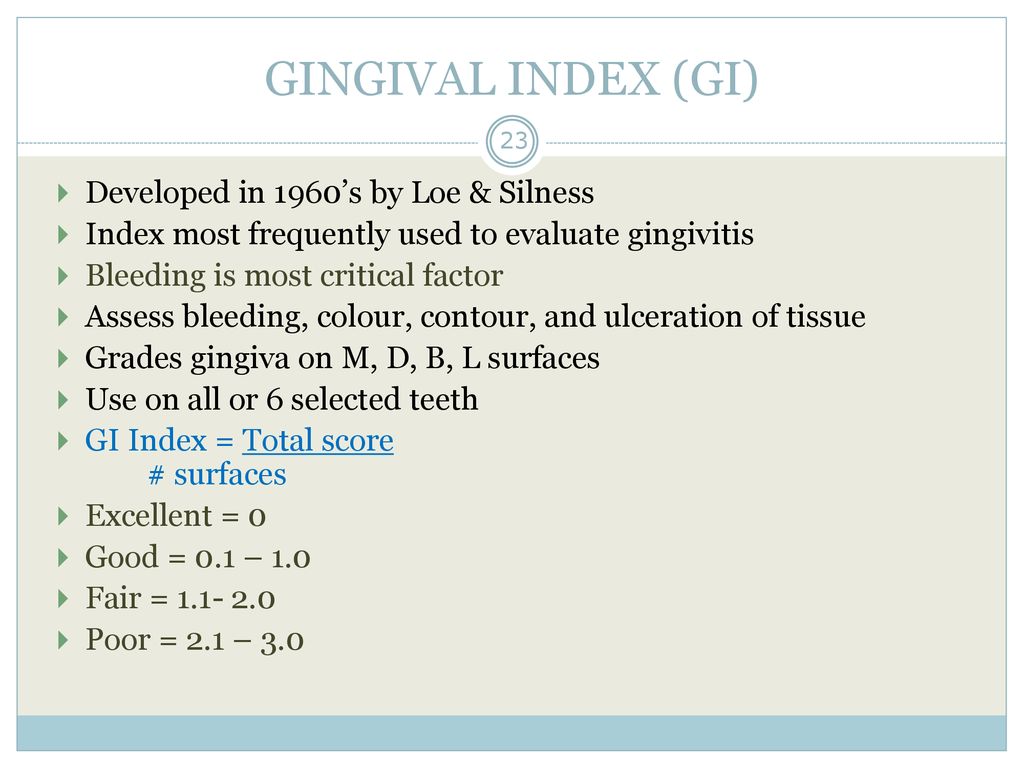


Excellent= 1

Good= 2

Fair= 3

Poor= 4

**Bleeding on probing (BOP)**

Presence or absence of bleeding after insertion of periodontal probe within the sulcus or pocket (Joss et al., 1994).

| **Bleeding Present** | Tick |
| --- | --- |
| Yes |  |
| No |  |

Yes=1

No=2

**Visible Plaque:**

Visible plaque will be identified by using a periodontal probe on the maxillary incisors as Yes and No

| **Plaque present** | Tick |
| --- | --- |
| Yes |  |
| No |  |

Yes=1

No=2

**Molar Classification:**

Modified angles classification will be used for the assessment of molar relationship

| **Molar Classification** | **Tick the box** |
| --- | --- |
| **Class I:** When the mesiobuccal cusp of the primary maxillary second molar occludes with the mesiobuccal groove of the primary mandibular second molar |  |
| **Class II:** When the mesiobuccal cusp of the primary maxillary second molar occludes with the interdental space between primary mandibular first and second molar |  |
| **Class III:** When the mesiobuccal cusp of the primary maxillary second molar occludes with the distobuccal groove or distal surface of the primary mandibular second molar |  |

Class I=1

Class II=2

Class III=3

| **Caries Risk Assessment Form** | | | |
| --- | --- | --- | --- |
| Patient information | | | |
| **Disease Indicators** | YES = CIRCLE | YES = CIRCLE | YES =  CIRCLE |
| Visible cavities or radiographic penetration of the dentin |  |  |  |
| Radiographic approximal enamel lesions (not in dentin) |  |  |  |
| White spots on smooth surfaces |  |  |  |
| Restorations last 3 years |  |  |  |
|  |  |  |  |
| **Risk Factors (Biological predisposing factors)** |  |  |  |
| Visible heavy plaque on teeth |  |  |  |
| Frequent snack (> 3x daily between meals) |  |  |  |
| Deep pits and fissures |  |  |  |
| Recreational drug use |  |  |  |
| Inadequate saliva flow by observation |  |  |  |
| Saliva reducing factors (medications/radiation/systemic) |  |  |  |
| Exposed roots |  |  |  |
| Orthodontic appliances |  |  |  |
|  |  |  |  |
| **Protective Factors** |  |  |  |
| Lives/work/school fluoridated community |  |  |  |
| Fluoride toothpaste at least once daily |  |  |  |
| Fluoride toothpaste at least 2x daily |  |  |  |
| Fluoride mouthrinse (0.05% NaF) daily |  |  |  |
| Fluoride varnish in last 6 months |  |  |  |
| Chlorhexidine prescribed/used one week each of last 6 months |  |  |  |
| Xylitol gum/lozenges 4x daily last 6 months |  |  |  |
| Calcium and phosphate paste during last 6 months |  |  |  |
|  | **HIGH** | **MODERATE** | **LOW** |
| CARIES RISK ASSESSMENT (CIRCLE): **HIGH, MODERATE, LOW**  **(EXTREME RISK= HIGH RISK + SEVERE SALIVARY GLAND HYPOFUNCTION)** | | | |

SPSS code High risk=1, Moderate risk=2, Low risk= 3
